# End‐of‐life priorities of older adults with terminal illness and caregivers: A qualitative consultation

**DOI:** 10.1111/hex.12860

**Published:** 2019-01-06

**Authors:** Ebony T. Lewis, Reema Harrison, Laura Hanly, Alex Psirides, Alexandra Zammit, Kathryn McFarland, Angela Dawson, Ken Hillman, Margo Barr, Magnolia Cardona

**Affiliations:** ^1^ Faculty of Medicine School of Public Health and Community Medicine University of New South Wales Sydney New South Wales Australia; ^2^ SWS Clinical School The Simpson Centre for Health Services Research University of New South Wales Sydney New South Wales Australia; ^3^ Department of Intensive Care Medicine Wellington Regional Hospital Wellington New Zealand; ^4^ University of Otago Wellington New Zealand; ^5^ Thomas Holt Aged Care Sydney New South Wales Australia; ^6^ Cunningham Centre for Palliative Care Sacred Heart Health Service St Vincent's Health Network Sydney New South Wales Australia; ^7^ Faculty of Health The Australian Centre for Public and Population Health Research University of Technology Sydney Sydney New South Wales Australia; ^8^ Intensive Care Unit Liverpool Hospital Sydney New South Wales Australia; ^9^ Centre for Primary Health Care and Equity Faculty of Medicine University of New South Wales Sydney New South Wales Australia; ^10^ Centre for Research in Evidence‐Based Practice Faculty of Health Sciences and Medicine Bond University Gold Coast Queensland Australia; ^11^ Gold Coast Hospital and Health Service Gold Coast Queensland Australia

**Keywords:** care priorities, end‐of‐life, family caregivers, older adults, qualitative study

## Abstract

**Background:**

As older adults approach the end‐of‐life (EOL), many are faced with complex decisions including whether to use medical advances to prolong life. Limited information exists on the priorities of older adults at the EOL.

**Objective:**

This study aimed to explore patient and family experiences and identify factors deemed important to quality EOL care.

**Method:**

A descriptive qualitative study involving three focus group discussions (n = 18) and six in‐depth interviews with older adults suffering from either a terminal condition and/or caregivers were conducted in NSW, Australia. Data were analysed thematically.

**Results:**

Seven major themes were identified as follows: quality as a priority, sense of control, life on hold, need for health system support, being at home, talking about death and competent and caring health professionals. An underpinning priority throughout the seven themes was knowing and adhering to patient's wishes.

**Conclusion:**

Our study highlights that to better adhere to EOL patient's wishes a reorganization of care needs is required. The readiness of the health system to cater for this expectation is questionable as real choices may not be available in acute hospital settings. With an ageing population, a reorganization of care which influences the way we manage terminal patients is required.

## 1 INTRODUCTION

Increased use of emergency services and hospitalizations among older people who are dying^1^ often includes intensive procedures^2^ that can prolong suffering and are too late to be of benefit.^3^, ^4^ Evidence that patients or families has been consulted regarding their preferences for future care and how this consultation has occurred is limited but critical to the provision of appropriate end‐of‐life (EOL) care.^5^

Older patients and their families are usually provided with information about hospital‐based treatment options^6^, ^7^ regardless of whether they wish to spend their last days in an acute care hospital.^8^, ^9^ You et al^10^ report that patients and families lack understanding of the implications of life‐sustaining treatments, with Wilmott and colleagues^11^ finding that a patient's substitute decision makers do not always act in the patient's best interest. As a result, a patient's wishes may not be known or honoured. Additional factors that can further complicate EOL decision making are as follows: the low public awareness^12^; cultural values affecting care preferences at the EOL;^13^, ^14^ family denial of the patient's prognosis;^15^ potential cognitive impairment in old age;^16^ conflicting family pressures;^17^, ^18^ and the level of a doctor's professional expertise in communicating a terminal prognosis sensitively.^19^

Health professionals providing quality EOL care across all health services must have an understanding of the family and patient's perception of what is appropriate and contributes to high‐quality care, and what constitutes a “good death.”^20^, ^21^, ^22^ This information is necessary to fully inform clinical and other support staff providing EOL health services. Existing data that demonstrate the use of medically inappropriate treatments at the end‐of‐life and the importance of engaging in advance care planning may assist to inform more honest end‐of‐life discussion.^23^, ^24^

Research to date on EOL care has been predominantly conducted in the cancer realm.^25^ With more people dying from diseases of ageing,^26^ this research, although informative, does not take into consideration the EOL trajectory of other terminal conditions. The Australian government recognizes the importance of providing high‐quality EOL care^27^ and developing guiding principles and essential elements for the provision of safe and high‐quality EOL care.^28^ Despite the Australian government's support for EOL care, a recent Australian‐based study found that only fourteen per cent of non‐cancer patients in the last year of life, with irreversible conditions which were considered amenable for palliative care, received specialist palliative care compared to more than two‐thirds of cancer patients.^29^ These non‐cancer patient conditions included the following: heart, renal and liver failure; COPD; HIV/AIDS; dementia; and Motor neurone, Parkinson's and Huntington's disease.

This study aimed to determine older terminally ill patients and caregivers’ priorities, perceptions and appropriateness in EOL care.

### 1.1 Objectives

1. Define current consumer priorities in EOL care for older terminal patients, their caregivers
2. Elucidate the main components of “quality” at EOL
3. Explore the perceived impact of treatment for terminal illness on the individual and their caregivers
4. Identify the important health service factors for quality EOL care

## 2 METHODS

### 2.1 Sample frame—Consumer EOL advisory group

The sample frame was members of the UNSW consumer EOL advisory group, established to identify priority concerns to inform on the public perspective of our research projects. Between November 2015 and March 2016, a call for older adults/carers of older adults to participate in the UNSW consumer EOL advisory group was undertaken through advertisements in academic and hospital/aged care networks and by word of mouth. The UNSW consumer EOL advisory group membership eligibility included the following: direct experience of health services for advanced chronic illness including terminal care either for a relative, friend or themselves; or experience in providing physical and social aspects of care for frail terminal older adults and/or their relatives towards their EOL; or commitment to the concept of improving the EOL experience for themselves or others. Those who were interested in becoming a member of the consumer advisor group responded via email and/or telephone. A total of 37 people, mostly aged over 60 years, joined the consumer EOL advisory group. However, it also included younger adults (30‐49 years) who informally cared for older people. Consumer group members attend a maximum of four consultations per year, with participation for each consultation voluntary. This study reports result from the Round 3 consultations.

### 2.2 Sample and data collection

All of the consumer EOL advisory group members were invited to participate in Focus Group Discussions (FGD) or In‐depth Interviews (IDI). A total of 24 (65%) agreed to participate. Ninety‐minute FGDs were conducted on the same day in April 2016 and 90‐minute (IDI) were undertaken during the month of June 2016. All FGDs comprised both terminally ill patients and caregivers in each group. One member of the study team (EL) conducted all IDI with patients being at home, which were necessary to capture perspectives of those who were unable to participate in FGD. This was due to geographical difficulty, as 30% of the Australian population reside outside major cities^30^ or due to the participant's poor physical health from their terminal condition which impacted on their mobility and transport capacity. Members of the study team developed the FGD guide which included four main topics EOL care, quality of life factors, family impact and health‐care provision (Appendix S1). The IDI guide that reflected the FGD topic was also generated for those who lived away from the city where the study was conducted or for those volunteers who were too ill to attend the focus groups. Written consent was obtained from each participant. Three study team members with either a psychology (RH) or a nursing background (EL, LH), who were trained in qualitative methods, facilitated the three 90‐minute FGDs in a private meeting room on a University campus. The facilitator guided participants through each of the topics. The FGDs were audio‐recorded and written notes were also taken by the team members. One team member (EL) was present for all FGDs and listened to the transcripts from all FGDs to ensure consistency. EL conducted all IDI using the agreed guide.

### 2.3 Data analysis

The audio‐recorded qualitative data were transcribed verbatim and managed using NVivo software (version 11 QSR, International Pty Melbourne, Victoria, Australia).

Thematic content analysis was used to elicit themes from participants regarding whether quality or length of life was most valued, attributes of good quality living, the effect of EOL involvement on caregivers and their experiences and expectations of health professionals providing this care. Two team members (RH, EL) undertook the thematic analysis,^31^ initially repeatedly reading the transcripts and then labelling the text in the NVivo software. Each team member then independently grouped labels into related themes around consistent or divergent issues arising. These researchers held iterative discussions in which they reflected on the emerging themes^32^ and then refined the themes into a final set of agreed categories. A third researcher (LH) who observed the FGD independently reviewed the categories and themes within these for face validity.

## 3 RESULTS

The final sample included 24 participants, 17 females and 7 males. Eighteen participants attended FGDs and six had an IDI. Ten participants suffered from a chronic progressive or life‐limiting illness including Chronic Kidney Disease with multiple transplants (1); Advanced Parkinson's Disease (1); Breast Cancer (1); Heart failure (2); COPD and inoperable brain tumour (1); Motor Neuron Disease (1); Organic dementia (1); frailty (2). Fourteen participants were carers of those who had suffered a terminal condition and faced EOL decisions. The sample was ethnically diverse with 14 born in Australia and 10 born in the Mediterranean, Eastern Europe, South Asia, Middle East and American countries. Participants were predominately aged 60 years and older (20), and five subjects lived in rural/regional Australia.

**Figure 1:**
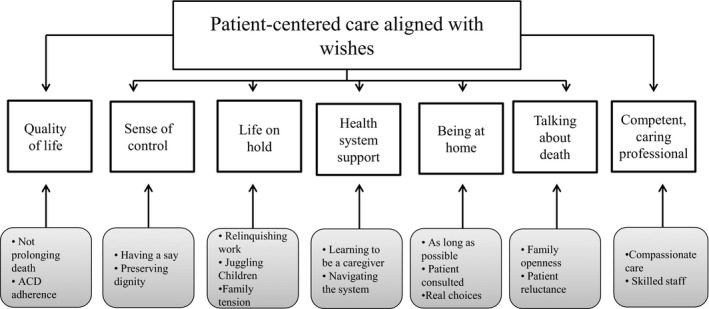
Themes on patient and family priorities at the EOL

Seven themes emerged from the analysis of the FGD and the IDI transcripts: *quality as a priority, sense of control, life on hold, need for health system support, being at home, talking about death and competent and caring health professionals*. An underpinning priority that fed through the six themes was knowing and adhering to *what the patient wants*. Figure 1 illustrates these themes and the factors within each theme that were assessed by the participants as being a priority for optimal EOL care. Each theme is also described below with supporting quotes.

### 3.1 Quality as a priority

Participants across the FGD and IDI agreed that a good quality of life was the most important consideration in EOL care; prolonging an uncomfortable existence was not the goal. Discontent with models of care that were not sensitive and responsive to quality of life considerations surfaced quickly in the discussion. Whilst rapid consensus was achieved regarding quality of life as the most important factor in EOL decisions, further exploration of the conceptualization of quality revealed nuanced understanding of what quality means to any given patient is critical to making EOL decisions. Prolonging life was, however, identified as an important consideration when there continues to be hope for treatment and when a patient is of a younger age.

I don't want to prolong my life at all. As long as I'm independent I'm quite happy but if I become reliant on other people I do not wish to live under the circumstance.

(Male, COPD and inoperable brain tumour)

I have no interest in the quantity of my life; I have every interest in the quality.

(Female, Organic dementia)

“So in prolonging life was there also quality of life during that prolonging? For me, that's a major question”

(Female, Caregiver)

Participants suggested that when making assessments and decisions about whether prolonging life is beneficial to the patient, understanding and adhering to the patient's wishes was seen as an essential part of personalised care. One example raised by many was the need for recognition of and adherence to advanced care directives when in place.

A year before that she had…gone to the solicitor and written that she didn't want any pharmaceutical, medical or surgical intervention. I came in the next day and she was being pumped full of antibiotics

(Female, Caregiver)

Part of the medical system is this giving of medications, keeping the medications on, ignoring directives like ‘Don't Resuscitate

(Female, Caregiver)

### 3.2 Sense of control

Participants did not define a discrete set of quality of life factors, but converged on the notion that a good quality of life is when an individual has control and can meet their own personal standards and expectations. These standards and expectations were perceived as dynamic throughout life and at the EOL as physical and cognitive abilities deteriorate.

So my quality of life description is ‐ and it's very personal ‐ up until I was 80 was to make 80 and it was going to give me the quality of life I wanted. After I turned 80 and then I'd had a fall this week…

(Male, Advanced Parkinson's Disease)

Quality of life when I think about myself is about having a say in my life and being able to have some self‐agency and to be able to have a say in what happens to me and to be able to have some capacity to direct things.

(Female, Motor Neuron Disease)

Good quality of life was consistently conceptualized as being able to do the things a person enjoys and maintaining their sense of self through these activities, or as the patient's ability to achieve their aspirations whatever those may be. Many examples were provided, particularly by carers, of activities and interests that, for their loved one, were markers of good quality existence.

He was a passionate music lover and that had been one of his great loves. So right up in fact to the moment that he was dying he was listening to his favourite.

(Female, Caregiver)

Loss of control was consistently identified by the participants as a loss of quality of life and linked to a perceived loss of dignity. Caregiver participants experienced distress at watching a loved one losing control of their thoughts and actions.

So by this time she gets to the nursing home, she's faecally incontinent; she's urinary incontinent; had to hoist her up on that thing to hose her down; you know absolutely awful.

(Female, Caregiver)

For someone with a mental disease, brain degeneration, as my husband has, who has no quality of life…everything has to be done for him… but we have to wait until there is another medical disease before he can be placed into palliative care.

(Female, Caregiver)

### 3.3 Putting family life on hold

Caregiver participants described the impact of their loved one's life‐limiting illness as putting their life on hold to care for another, making financial, career and personal compromises in order to do this. All of the participants who had experienced caring for a loved one at the EOL discussed common features of this caring role.

In the last year of my mother's life I had three fairly young children. I used to go at 6:30 am or 6:00 am to visit her so I could get home in time to take the kids to school. I was also at full time university…I did it somehow but it certainly impacted.

(Female, Caregiver)

It was enormously stressful because this is over a period of six years. My sister was working, I wasn't, but our parents lived separate from both of us so we had lot of the car driving, a lot of expense.

(Female, Caregiver)

“Juggling” family life and caring responsibilities was a challenge, with many participants highlighting the uneven distribution of caring responsibilities between family members. In some cases, this raised tensions between family members, and in some cases led to the process of agreeing roles.

“My sister and I are very different, but we negotiated the care really, really well, because we both acknowledged what our strengths were and we did them.”

(Female, Caregiver)

“I guess as the person who takes on the most of the caring role you sometimes feel a bit abandoned by the rest of your family because it's just presumed you will be there…”

(Female, Caregiver)

Carer participants described EOL care as emotionally difficult. Feelings of guilt, denial, distress and sadness were noted in addition to the physical and logistical challenges of caring for a loved one. Although caregiver participants sought to adhere to patients’ preferences regarding not prolonging their life, conflicting feelings occurred as participant caregivers also did not want to lose their loved one.

I think I've had both those feelings; wanting them to hang around, but also wishing them a speedy goodbye for their sake.

(Female, Caregiver)

### 3.4 Need for health system support

Both patients and caregivers identified challenges in navigating the health system, although these were conceptualized differently. For patients, attending appointments for care at the EOL could be challenging, with the difficulty of multiple visits to services that were not localized, compounded by poor mobility and the associated costs of transport.

I have to go to (X) hospital for several injections a couple of times per week and if for instance I went by taxi it cost me 100 dollars or more return but if I take public transport I have to take three different busses and a train and if everything went bad it could take me up to 6 hours in transport

(Male, COPD and inoperable brain tumour)

For caregivers, navigating the complex health system and learning to be a carer for the first time was physically and emotionally challenging. Respondents converged on the difficulties of liaising with multiple services and providers, trying to establish service availability and also gaining access. Navigating the system was particularly challenging whilst being a caregiver, and in some cases, working in professional employment alongside the caring role. The need for greater support system was noted by many during the discussion.

I would like to see them (caregivers) being formally supported in some way….supports people through what they're learning, because you're learning stuff. You know it's like this whole new world.

(Female, Caregiver)

I'd really like to see something in institutions where there was somebody who would help coordinate some kind of care amongst the people who care about this person

(Female, Caregiver)

### 3.5 Being at home (at EOL)

Participants agreed that it was generally preferable to stay at home for as long as possible. Consultation with health providers and choice regarding the location of their treatment and care was identified as important.

Dad was in palliative care and basically he didn't want to be there at all…one day mum went in and he said take me home….So he was home for a week and then passed away at home, but at least he met his wishes.

(Male, Caregiver)

The hospital is an alternative, I would never say the home is an alternative… the hospital has to be the last place on earth in any country that you would need to go simply to die.

(Female, Caregiver)

Yet caregiver participants identified cases in which this was not possible, such as when the person or their caregiver did not have the ability to provide the care required. In these cases, carer participants often reported a need for greater support from other social or community‐based services to facilitate care at home.

We knew we couldn't deal with it at home ourselves, but had there been other kinds of care, that would have been perfect. He would have still been in his own garden and done his own little pottering around the place as he always did

(Female, Caregiver)

She couldn't cope at home and she's gone into an old age home and that's much better all‐round than trying to cope at home.

(Male, Caregiver)

### 3.6 Talking about death

Openness about impending death, honesty and transparency between patients, their family and health‐care providers was viewed as important in ensuring appropriate, patient‐centred, EOL care.

Yet participants highlighted the difficulty of talking about death at every level; between patients, family, health professionals and at a societal level. Discussions about the EOL were identified as limited, lacking, too late and emotionally challenging, leading to a lack of sufficient understanding of each patient's wishes.

I think it's a bit the same as the culture, the kind of health culture, there's not a kind of a literacy in our community, in our society around death. It's not easily spoken about.

(Female, Caregiver)

Caregiver participants often described the reluctance of their loved one to talk about their deterioration and what they wanted. Some identified this as culturally influenced but the participants generally agreed that discussions about death were uncommon across cultures.

The doctor brought up the question of the directive to see whether to switch off the life support machine just in case … but she hated to talk about that. Plus with our cultural background they don't like to talk about it.

(Male, Caregiver)

She was actually very grateful for the way that the doctor spoke to her… and I think all of us that were in that chemo room with all the other women, we recognised that in him, that that's the way he operated and I think everybody appreciated that kind of honesty.

(Female, Caregiver)

### 3.7 Competent and caring health professionals

Participants identified that a consultative, patient‐centred care approach was critical. They stated that health professionals, who were compassionate, respectful, and ensured patient dignity, played a significant role in providing quality EOL care to patients and caregivers.

…Good relationships with the primary health team is what I think is absolutely essential…. …A good relationship someone who understands you and understands the family and who will work with other professionals….

(Female, Motor Neuron Disease)

She came in to find her with an oxygen mask on and we had said no resuscitation. The doctor said something like she won't need that anymore and walked out the door. That was how my sister discovered mum had died.

(Female, Caregiver)

## 4 DISCUSSION

The priorities for high‐quality EOL care identified by the study participants, who were caregivers of people who have had or are experiencing terminal conditions or patients who were suffering from a terminal illness themselves were as follows: quality as a priority, sense of control, how to manage life on hold, need for health system support being able to remain at home if possible, talking about death to know what patient wishes are, and having competent and caring health professionals. In particular, our data highlight the importance of knowing and adhering to patient's wishes (if known) when providing EOL care.

Our findings reinforce the call for patient‐centred care, that is, health care that is responsive to the preferences, needs and values of each patient^33^ regardless of whether the goals of care are curative and interventional or focused on a palliative approach. Our findings also support the need to include consumer voices in facilitating health service improvement.^34^

According to other research with terminal patients and their caregivers, priorities for high‐quality EOL care have included the following: the need for professional communication, honest consultation on preferences, respect for patient dignity, support in navigating the health system, control in decision‐making, consideration of the burden on family life, and access to skilled health practitioners who are good communicators.^35^, ^36^, ^37^, ^38^ A systematic review in 2015 of quantitative studies in Canada, US and the UK aiming to find the most important aspects of inpatient EOL care of palliative patients and their family found similar results to our study in Australia.^23^ Since that review, we identified two relevant qualitative studies which included people in the United Kingdom with dementia^39^and caregivers of people with advance cancer in Australia.^40^ Despite the disease‐specific study populations, there were similarities between our study which included caregivers and terminally ill who were suffering a broad range of terminal conditions and the themes identified in the dementia and cancer populations which included the following: being at home (or a home‐like environment) at the EOL; competent and skilled health professionals at the EOL; being comfortable as important components of good EOL care;^39^ and the readiness of caregivers to engage in EOL discussions.^40^

In our qualitative study, participants strongly favoured higher quality supportive care as opposed to prolonging life at all costs, which is consistent with an Australian survey finding that the majority of older adults believe quality of life is “paramount.”^14^ However, participants reported that when making decisions about prolonging life there was inconsistency in the degree to which patient and family had been involved in EOL care contexts. Specifically, participants reported that, health professionals did not always follow patient wishes and advance directives. Factors have been identified before as contributing to this limited involvement of health consumers such as a lack of clear written documentation to facilitate decision making at the time of admission;^41^ clinician‐consumer divergent opinion on the prognosis or interpretation of the words “terminal”;^42^ pressure from relatives;^43^ and the relationship between the health professional, patient and caregiver.^44^

The preference to be at home for their EOL care reported in our study is consistent with Foreman and colleagues population survey in Australia over a decade ago that reported 70% of Australians preferred home as a place of death if suffering from a terminal illness.^45^ Yet as Pollock^46^ (2015) identifies, there are difficulties with regard to the management of severe symptoms away from hospitals. Our participants were aware that in many cases, home death was not possible due to the challenges of an EOL context, including the increasing care needs as the person deteriorates, the patient‐provider relationship, the role and feelings of family or friends who were caregivers, and the availability or feasibility of the health system to provide particular services. Our caregivers also expressed the need for system support to navigate the health‐care system for loved ones which they often felt unprepared for. Jeff's^47^ et al (2017) found similar results in Canadian caregivers of older adults that reported complexity and challenges navigating the health system during interfacility care transitions.

Recent evidence indicates that the use of early community‐based palliative care referrals is associated with a reduction in hospital emergency department use in patients with dementia in the last year of life^48^ and in reducing cancer patients’ transfer to acute hospitals in the last 90 days before death.^49^ Consistent with our consumers’ preferences, the provision of a palliative care approach in any setting including home‐based has shown to enhance satisfaction and increase the likelihood of death at home^50^ as well as being more cost‐effective.^51^ However, existing models of EOL care for frail older adults would require significant changes to be implemented according to patient's wishes if many prefer to die at home.^52^ As is described in the national consensus statement for safe and high‐quality EOL care, with an ageing population, a reorganization of care and the way we manage terminal patients is required.^28^

Despite recommendations on addressing EOL care outside of acute care settings that respect patient preferences to die at home and support informal caregivers,^53^ many patients still spend their last days in an acute hospital.^54^ Most western health systems do not appear ready for widespread community supported palliative care, as illustrated by previously reported barriers; the absence of skilled EOL workforce outside specialist health‐care facilities;^55^ substantial out‐of‐pocket costs of residential aged care;^56^ and the lack of infrastructure to meet demand in countries with universal health care has resulted in long waiting lists for eligibility assessment.^57^, ^58^ Failures in organisations to support advanced care planning in partnership with patients, along with ineffective communication will continue to prevent optimal and safe EOL care for the frail older adults.

This qualitative study involved public consultation and representation of views including those of different ages, ethnicity and experience of health care. Involving older people as advisors has shown to enhance the relevance of health services research.^34^ The information collected in our consultation covered recent experiences in the health system and home settings and is of relevance for clinicians and health service planners. IDI supplemented the FGD findings with extensive details from less physically mobile health service consumers.

A possible limitation of our study is that the majority of participants were females and caregivers. However, as females are often the informal caregivers of chronically ill patients^36^, this may in fact be representative of the reality of informal caregivers. The fact that we only conducted three FGD could also be considered as a limitation; however, saturation was rapidly achieved even with three FGD. While our study confirmed that consulting patients and families about this sensitive topic is feasible in Australia, this consultation did not happen at a time of acute medical crisis. It could be argued that our study did not take into account patient and family preferences at those critical times, as studies have shown that preferences can change over time depending on a person's state of health.^59^ However, we believe the views our participants are further enriched by the ability for retrospection without the influence of an acute emotion.

## 5 CONCLUSIONS

This consultation identified priorities and preferences by consumers through their experiences of the delivery of EOL care. It confirmed that the health system still faces two persistent barriers to the delivery of satisfactory, safe and high‐quality end‐of life‐care for consumers: shortage of strategies to address the unmet needs of terminally ill older adults and caregivers, and the need for health professionals to deliver more skilled communication incorporating personal values. Unfounded perceptions that patients and carers are not open to EOL conversations or shared decisions on goals of care at the EOL such as limitations of treatment need to be revisited. With an ageing population, a reorganization of care to optimize the way we manage terminal patients is overdue. The readiness of patients and families for proactive engagement in advance care planning represents an opportunity to slow down unsustainable public demands for aggressive care and promote effective communication to prevent suboptimal and unsafe EOL care for the frail older adults.

## ETHICAL APPROVAL

Ethics approval was granted by the University of New South Wales Human Research Ethics Advisory Panel (HREAP # HC16159).

## INFORMED CONSENT

Informed consent was obtained from all individual participants included in the study.

## ACKNOWLEDGEMENTS

We thank members of the consumer advisory group for their insightful contributions to this research. Mr Chhorvoin Om provided initial assistance with concurrent thematic interpretation of data from one of the focus groups.

## CONFLICT OF INTEREST

The authors declare they have no conflict of interest.
