# Supplementary material for: End‐of‐life priorities of older adults with terminal illness and caregivers: A qualitative consultation
Source: Health Expect. 2019 Jan 6;22(3):405–14. doi: 10.1111/hex.12860 (PMC6543262; doi:10.1111/hex.12860)
Supplement: Supplementary file 1 [file HEX-22-405-s001.docx]

**Appendix. Guide for Focus Group Discussion and In-depth Interviews**

*Good afternoon, my name is ________ and these are my colleagues….(names)……… Thank you for contributing further to our understanding on aspects that may affect consumers in the delivery of care at the end of life. As you know, this is Round 3 of the consultation and the purpose of this session is to refine our findings based on your views on end-of-life care preferences and what constitutes wellbeing, as derived from the previous two consultations. For the next hour-and-a-half we would like you to exchange experiences, perceptions and learnings about your experiences of participating in decisions for patients/older relatives who are dying or being a caregiver or a healthcare consumer in the context of terminal or chronic advanced illness.*

- *Let me remind you there are no right or wrong answers and the content of all discussions held here will be treated as confidential by everyone.*
- You can choose not to use the real names of people if you use a story to explain your ideas
- Try not to gossip after the group
- Let everyone have a chance to speak and be respectful.
- *Given the sensitive topic area, there is the potential for group members to experience feelings of distress. As you are aware of the purpose of this research and volunteered to openly discuss, we do not anticipate that this will occur too often but should this happen to you, other group members including the investigators can support you on the day, and we have a list of local services where you could find assistance free-of-charge following this meeting.*

*Our reports or manuscripts will summarise comments arising from this discussion but individual participants will not be identified.*

*We have obtained consent from you before this session but remember you don’t have to participate in all questions if you do not feel comfortable or prefer just to talk rather than write, or even if you prefer not to take part.*

Researcher to:

- Ice-breaker/refresher introductions of all participants (as some know each other from previous meetings)
- Check that it is OK with everyone to record the session or part of it
- Go through study information and address questions

*I will introduce each topic area with the opening questions shown and then we will discuss as a group and I will use follow up probes to steer the discussion where necessary. Let’s start with*

**Topic 1: Priorities in EOL care**

- To what extent is end of life care about simply prolonging the length of a person’s survival?
  - How important is their comfort?
  - How important is their quality of life?

**Topic 2: Quality of life factors**

- When you think about quality of life what kinds of things are important to you for a good quality of life?
  - Probes – types of activities that can be done.

**Topic 3: Family impact**

- In what ways does a patient’s health status impact on family life – for example the family day to day routine?
- To what extent is it important that the family agree the appropriate course of care – what kinds of challenges does this raise?

**Topic 4: Healthcare provision**

- What factors are important in the provision of services in end of life care? – provide examples of factors through probes:
  - Probes – honesty, location (home/hospital), cost/affordability, meeting cultural/traditional beliefs.
  - Are there real alternatives to hospital end-of-life care for people living outside large cities? **(This additional question was asked to participant outside large cities in the in-depth interviews**)

*Are there any further recommendations, comments or questions before we close the session?*

**CLOSING**

***Brief oral summary*** *(by facilitator) --Is this an adequate summary? Have we missed anything?*

***Thank you*** *for your time and for sharing your ideas with us today and for being so considerate and respectful of one another.*

***Please remember the ‘no gossip agreement’*** *and that you can talk* *about the Questions and your answers, but not other people or what they say. You can also talk to me or Dr MCM if something said in the group bothers you.*

***So what happens next?***

*After we’ve spoken to all the Advisory Group members, we’ll put everyone’s ideas together as a semi-final draft of the definitions and instruments we are developing. We’ll look at the similar / different ideas and see how today’s comments can enhance what we already know from previous consultations and put these in a report to discuss at the next and final round of consultation. We will be refining some of the patterns in this information and will give you feedback. After this we will test the concepts and instruments in hospital patients as part of another study.*

*I’m sure at the end of the consultation rounds your combined views will be really helpful to other patients, families and healthcare professionals working in this field.*
